# Delay in treatment seeking and associated factors among suspected pulmonary tuberculosis patients in public health facilities of Adama town, eastern Ethiopia

**DOI:** 10.1186/s12889-019-7886-7

**Published:** 2019-11-14

**Authors:** Tirusew Maru Wondawek, Musa Mohammed Ali

**Affiliations:** 1 Oromia regional health bureau, Adama Public Health Research and referral Laboratory center, Adama, Ethiopia; 2 College of Medicine and Health Sciences, School of Medical Laboratory Science, Hawassa University, Hawassa, Ethiopia

**Keywords:** Delay in treatment of tuberculosis, Risk factors, Adama, Ethiopia

## Abstract

**Background:**

In low-income countries, delays in treatment seeking among tuberculosis patients contribute to easy transmission and high prevalence of tuberculosis.

**Objective:**

The aim of this study was to determine the magnitude of delays in treatment-seeking and risk factors among pulmonary tuberculosis suspected patients in health facilities located in Adama, Ethiopia.

**Method:**

A health-facility based cross-sectional study was conducted at Adama from December 20, 2015, to March 1, 2016, among 598 tuberculosis suspected patients. Data was collected from all study participants on the same day of tuberculosis diagnosis using a structured questionnaire. Epi-Info 3.5.3 and Statistical package for the social sciences (SPSS) version 16.0 were used for data entry and analysis respectively. A bivariate and multivariable regression model was used to investigate the association between delay in seeking-treatment and various factors. Odds ratio with 95% CI and *P*-value < 0.05 were considered as cut off point to measure the strength and significance of the association.

**Results:**

Among 598 pulmonary tuberculosis suspected patients, 79 (13.2%) were smear-positive. Among smear-positive participants, 61(77.2%) delayed seeking treatment and 275 (46%) patients delayed seeking treatment for > 30 days. The following factors were significantly associated with a delay in seeking treatment: female sex OR = 1.57, 95% CI (1.14, 2.18), low monthly income OR = 1.45, 95% CI (1.05, 2.01), lack of knowledge regarding tuberculosis OR = 1.67, 95% CI (1.13, 2.48), and cure rate of tuberculosis OR = 1.836, 95% CI (1.25, 2.69).

**Conclusion:**

Nearly half of pulmonary tuberculosis suspected patients delayed seeking treatment in our study area. Female sex, low income, family size of five and greater, no knowledge about tuberculosis and cure rate were factors contributing to delay in treatment-seeking among suspected tuberculosis patients.

## Background

Although Tuberculosis (TB) is preventable and curable, it remains a major cause of morbidity and mortality in many countries. According to a global TB report in 2017, ten million people were infected with TB and 1.6 million died from the disease [1]. Globally, TB incidence is falling by 2% per year but to reach the 2020 milestones of the ‘End TB Strategy’ the annual decrease should be 4–5% [1]. Through TB diagnosis and treatment between 2000 and 2017 about 54 million lives were saved [1].

In 2014, Ethiopia identified 119,592 new TB cases and enrolled 597 drug-resistant cases [2]. Most of TB cases were identified in the productive age group [2]. According to report from Ministry of Health (MOH) hospital statistics indicates, TB is the third cause of death in Ethiopia [3]. Health indicators in 2009 revealed a 36.6% case detection rate in the Oromiya regional state with an estimated 48,310 smear-positive cases [3]. As most TB affected individuals belongs to the productive age group, this could affect socio-economic development of the country [3].

Although Ethiopia has recognized TB as a major public health problem and adopted the Direct Observed Therapy (DOT) program in 1992, the disease is still a major public health problem. The main obstacle in controlling TB is to improve the detection of sputum smear-positive TB cases which depends on passive case findings and self-presentation to a health facility. Delay in TB diagnosis increases risk of death and prolong the period of time they are infection [4–6].

Delays in seeking treatment among TB patients are a major problem in most low-income countries including Ethiopia, where less than half the estimated sputum smear-positive pulmonary TB cases are detected. Income, knowledge, and availability of health care facilities are among some factors that contribute to delays in health seeking among TB patients [6, 7].

Ethiopia is a federal state, where programming and policy implementation occur at different levels. This includes the Federal MoH (FMoH), Regional Health Bureau (RHB) levels, health facility level, and TB diagnostic laboratories at multiple levels. The functions of the FMoH are setting national policy and standards, providing national guidance, and technical and financial support to RHBs. At the sub-national level, the RHB TB and Leprosy program is managed by TB unit/case teams under health promotion and disease prevention core-processes in FMoH [8].

Health posts, health centers, hospitals and private health facilities deliver TB prevention and care service. In Ethiopia TB diagnosis and treatment is provided for fee. In TB diagnostic laboratories at different levels, diagnostic laboratories are coordinated and assisted by the Ethiopian Public Health Institute (EPHI). TB laboratory services are packaged and delivered at three levels of the health care delivery system: National referral laboratory; Regional referral laboratories and peripheral laboratories at health centers and hospital laboratories including private health sectors [8].

Although there are some data on delay in seeking treatment among TB patients in Ethiopia, the main reasons for this delay were not clearly identified in Adama town. Identifying the magnitude and factors which are associated with patient delay in treatment-seeking will help to improve TB control by increasing case findings, provide early treatment and reduce the infectious duration in the community. These findings could improve patient management through early initiation of effective treatment. The community Health Bureaus and health facilities would also benefit from this study by recognizing the challenges and potential gaps in patient treatment seeking behaviors and identifying whether further studies in the area are warranted. Therefore, the aim of this study was to assess the delays in treatment-seeking and associated factors among suspected pulmonary TB patients in Adama town, Eastern Ethiopia.

## Methods

### Study design and study area

A cross-sectional study was conducted in health facilities which give TB diagnosis and treatment service in Adama town from December 20, 2015, to March 1, 2016. Adama is located in Oromia regional state. It is found 100 km due east of Addis Ababa, Ethiopia. The total population of Adama is 220,212. It is located at 8^o^33’N39^o^16’E/8.55^o^N39.27°E at an elevation of 1712 m [9].

### Study population

All patients who visited outpatient departments of public health facilities (Adama hospital Medical College, Geda health center, and Adama health center) found in Adama town were the source population. For this study, we recruited pulmonary TB suspected patients for whom sputum examination was performed. To recruit participants we used convenience sampling technique.

### Sample size

For this study, the sample size was calculated by taking a 53% prevalence of delay in treatment seeking reported from other place [6] with 95% Confidence level, 4% margin of error and using a single population proportion formula. The total sample size calculated was 598. We also calculated the sample size for the risk factors; however, since the calculated sample sizes for risk factors are less than 598 we took 598 as final sample size.

### Operational definition

Delay in treatment-seeking was defined as: the time between the onset of TB symptoms and patients first help-seeking action at health facilities. We assumed the patient was delayed if the patient sought medical care at health facilities after 3 weeks of the onset of TB symptoms [10].

Suspected Pulmonary Tuberculosis was define as: a patient that has a TB sign and symptoms such as fever, weight loss, night sweet, appetite loss and cough for 2–3 weeks [10].

### Data collection procedure

A structured questionnaire, which was adopted from a document prepared by the TB CARE II project with some modification [11], was used questionnaire to collect socio-demographic data from the study participants. In the questionnaire we included questions related to socio-demographic and economic characteristics, the interval between onsets of symptoms and first health provider consultation, and reasons for the delay in care-seeking. Data was collected before specimen sample collection after informed consent was obtained.

After socio-demographic and economic data was collected, participants were requested to provide three sputum specimens (spot, morning, and spot). Spot specimens were collected on the first day of visit, morning samples were collected early in the morning of the second day and the third specimen was collected after the morning specimen was submitted. Specimens were collected in a leak-proof and 50 ml screw cup containers. Study participants were advised to provide sputum as saliva is not suitable for laboratory examination. The AFB test result was collected participating health facilities. The test was performed after running internal quality control test [12].

### Data quality control

Supervisors and data collectors were trained on research tools and data collection procedures. The questionnaire was translated into the local language. A pre-test was performed to check consistency of the questionnaire and the time it took to conduct the interview. Supervision was provided throughout the data collection period by supervisors and the principal investigator to check the completeness and consistency of questionnaires.

### Data analysis

Complete pre-coded data was double entered into a computer using Epi-Info version 3.5.3, and transferred to SPSS version 16.0 after cross-checking and data cleaning. Tabulation, frequencies, proportion, and summary statistics were used to present the distribution of the study findings and to check missing values**.** Binary logistic regression was used to determine the association between dependent and independent variables. Variables that have a significant association with delay in treatment-seeking in binary logistic regression were further tested by multivariable model. Multivariable logistic regression was used to determine the relationship between several independent variables and a dependent variable. Odds ratio with 95% CI and *P*-value <0.05 were considered as cut point to measure the strength and significance of the association.

## Results

### Socio-demographic and economic characteristics

The response rate in this study was 100%. Out of the total of 598 study participants, 327 (54.7%) were females, 156 (26.1%) were in the 25–34 age group, 183 (30.6%) has completed secondary school, and 178 (29.8%) attended no formal education (Table 1).

**Table 1:** Socio-demographic and economic characteristics of the study participants in public health facilities of, Adama town, East Ethiopia, December 20, 2015, to March 1, 2016.(*n* = 598)

Variable	Frequency	%
Sex:		
Male	271	45.3
Female	327	54.7
Age in years:		
15–24	146	24.4
25–34	156	26.1
35–44	104	17.4
45–54	84	14.0
55–64	42	7.0
>65	66	11.0
Marital status:		
Single	132	22.1
Married	401	67.1
Divorced/Separated	65	10.9
Employment status:		
Yes	190	31.8
No	408	68.2
Educational status:		
No formal education	178	29.8
Primary	157	26.3
Secondary	183	30.6
Above secondary	80	13.4
Family size:		
1–4	283	47.3
>5	315	52.7
Relation with Head of HH		
Head of house hold	338	56.5
Spouse of head of house hold	82	13.7
Son/ Daughter	151	25.3
Other relation	27	4.5
Monthly income in Birr		
<650	296	49.5
>650	302	50.5

*HH* House hold

### Delay in treatment-seeking and prevalence of smear positive TB

Out of 598 suspected TB patients, 79 (13.2%) were smear positive and 275(46%) delayed seeking treatment. Of the smear-positive participants, 61(77.2%) delayed seeking treatment. Among the delayed participants, 109 (39.6%), 91(33.1%), 61(22.1%), 24(8.7%) and 5(1.8%) visited a health facility for treatment after 31–45, 46–60, 61–75, 76–90, and > 91 days of onset of illness. The median treatment-seeking period in the current study was 30 days. Of the delayed patients, 167(60.7%) were females. The majority of delayed participants 203(73.8) were married. Ninety-eight (35.6%) of delayed patients had no formal education.

### Factors associated with a delay in treatment seeking

In bivariate analysis study participants who commonly sought other treatment options, who lacked knowledge about TB, or who did not perceive that they can be infected by TB were COR = 2.84, 95% CI (1.05, 7.69), COR = 1.67, 95% (1.13, 2.48), and COR = 1.86, 95% (1.19, 2.9) more likely to delay seeking treatment respectively (Table 2).

**Table 2:** Factors associated with a delay in treatment seeking among study participants in public health facilities, Adama town, East Ethiopia, December 20, 2015, to March 1, 2016 (*n* = 598)

Variables	Patient delay		COR(95%CI)
	Yes n(%)	No n(%)	
Facility for TB treatment seeking			
Governmental	241 (87.6%)	290 (89.8%)	1.38 (0.69,2.73)
Other option	11 (4%)	19 (5.9%)	2.84 (1.05,7.69)
Private health facility	23 (8.4%)	14 (4.3%)	Ref
Knowledge about TB			
Yes	49 (17.8%)	86 (26.6%)	Ref
No	226 (82.2%)	237 (73.4%)	1.67 (1.13,2.48)
Susceptible to TB			
Yes	34 (12.4%)	67 (20.7%)	Ref
No	241 (87.6%)	256 (79.3%)	1.86 (1.19, 2.9)
Distance from health facility			
<10 km	182 (66.2%)	213 (65.9%)	Ref
>10 km	93 (33.8%)	110 (34.1%)	0.99 (0.70, 1.39)
TB can be cured			
Yes	195 (70.9%)	264 (60.4%)	1.00
No	80 (29.1%)	59 (39.6%)	1.84 (1.25,2.69)

TB: Tuberculosis, COR: Crude Odd Ratio, AOR: Adjusted Odd Ratio, Ref: Reference

In a bivariate analysis females were 1.57 times more likely to delay treatment seeking than males with OR = 1.57, 95% CI (1.14, 2.18). Married participants were 3.14 times more likely to delay seeking treatment. Participants who had family members of five and greater were 1.68 times more likely to delay seeking treatment than those who had less than five OR = 1.68, 95% CI (1.21, 2.33) (Table 3).

**Table 3:** Factors independently associated with a delay in treatment-seeking in public health facilities Adama town, East Ethiopia, December 20, 2015, to March 1, 2016 (n = 598)

Variables	COR(95% CI)	AOR(95% CI)
Sex:		
Male	Ref	Ref
Female	1.57 (1.14,2.18)	1.58 (1.11,2.236)
Marital status:		
Single	2.26 (1.16,4.37)	2.53 (1.37,4.67) 3.4 (1.58,7.34)
Married	3.14 (1.73,5.71)	Ref
Divorced/Separated	Ref	
Employment Status:		
Yes	1.48 (1.05, 2.09)	1.62 (1.12,2.34)
No	Ref	Ref
Family size:		
1–4	Ref	Ref
>5	1.68 (1.21,2.33)	1.76 (1.24,2.50)
Relation with Head of HH:		
Head of HH	Ref	Ref
Spouse of HH	0.74 (0.45,1.2)	0.85 (0.50,1.45)
Son/daughter	0.60 (0.40,0.89)	0.50 (0.29,0.88)
Other relation	0. 91 (0.42,2.01)	0.63 (0.27,1.48)
Monthly income in ETB		
<650.00	1.45 (1.05,2.01)	1.53 (1.07,2.18)
>650.00	Ref	Ref
Knowledge about TB		
Yes	Ref	Ref
No	1.67 (1.13,2.48)	1.86 (1.17,2.96)
TB can be cured		
Yes	1.00	1.00
No	1.836 (1.25,2.69)	1.66 (1.13,2.46)

*TB* Tuberculosis, *AOR* Adjusted Odd Ratio, *COR* Crude Odd Ratio, *ETB* Ethiopian Birr, Ref: Reference

In multivariate analysis delay in seeking treatment among suspected TB patients was significantly higher among females OR = 1.58, 95% CI (1.11, 2.236), among married participants, AOR = 3.4, 95% CI (1.58, 7.34), among participants who had a family size of five and greater were AOR = 1.76, 95% CI (1.24, 2.50), and among those who had a monthly income of less than 650.00 Ethiopian birr (ETB), AOR = 1.53, 95% CI (1.07, 2.18) (Table 3).

## Discussion

### Delay in treatment seeking and prevalence of smear positive TB

This study revealed a substantial delay in treatment seeking behaviors among TB suspected patients in Adama town. In our study, the prevalence of delay in treatment seeking was 46%. This rate is concordant with a report from Tigray Region, Ethiopia (53%) and Cambodia [13, 14]. In this study, the median patients’ delay was 30 days which is similar to the study conducted in Gojam, Ethiopia [15] Tanzania [16], and Italy [17]. However, the prevalence of delay in seeking treatment among TB patients we found is lower than studies conducted in Southern parts of Ethiopia (65%) [5], Wollo, Ethiopia (62.3%) [18], North Showa, Ethiopia (59.9%) [19] and Bale Zone, Ethiopia (96%) [20]. Unlike the current study, a short median patients’ delay (17 days) was reported from Addis Ababa, Ethiopia [21]. The difference observed can possibly be explained by study period, residence of the study participants (rural versus urban), socio-demographic and economic background. The prevalence of smear positive TB identified in our study (13.2%) is in agreement with report from North West of Ethiopia (10.4%) [22]; however, it is higher than prevalence of TB reported from Addis Ababa, Ethiopia (5%) [23]. On the other hand, the prevalence of smear positive TB we found is lower than report from country level prevalence of TB (19%) [2] and report from Northwest of Ethiopia (15.2%) [24]. The difference observed can be due to differences in study setting, characteristics of study participants, and laboratory method used.

### Gender

The proportion of delay in seeking treatment was significantly high among female study participants. This finding is consistent with studies conducted in Nigeria [25] and Tanzania [16]. However, a study from India showed that male study participants waited longer time than females to seek treatment [26]. This may be due to the socio-cultural difference between the two countries. Females’ tendency to delay in seeking treatment in our study could be attributed to their limited decision making power, engagement in domestic work, low level of education, and high rate of unemployment [25]. Moreover, socio-economic and cultural position of women may influence their decision in the society that could directly or indirectly affect their health needs [27].

### Marital status

The proportion of delay in seeking treatment was high among married participants. This finding is in line with the study conducted in Cambodia [14]. Participants who were not married have more opportunity to visit health facilities earlier than married participants. This can be due to married individuals having additional responsibilities and may not have time to seek treatment as early as possible. A study from Cameron also revealed that high proportion of individuals who are the main income earner for the family are more likely to delay in seeking treatment. This indicates that a person who is in charge of the family (source of income) wait longer before he/she visits health facility to avoid interruption of income [28]. In contrast to our study, a report from Tanzania indicated no association between marital status and delay in treatment-seeking [29]. Unlike our study, married participants from Tanzania in spite of their workload and responsibilities respond to their health problem the same as single and divorced participants. The difference observed could be explained by differences in the level of awareness about the disease.

### Economy

In this study we have identified low monthly income as an important factor that contributes to patients delay in seeking treatment. As diagnosis and treatment is free for TB patients in Ethiopia, money is required mainly for transportation, accommodation and other related activities. Since wages are low, individuals with low income need to work long hours that may not allow them the time to respond to their health needs in a timely manner. Moreover hey may not have sufficient money for transportation and accommodation and are therefore more likely to visit health facility only whenever they are critically ill. Among sub-Saharan African countries, 65% of the population of Ethiopia earns less than one United States Dollar per day [30]. Poverty is one of the most important factors that affect early treatment. Similar to our study, reports from Bangladesh [31], Laos [32] and Nepal [33] have documented income as an important factor for delay in seeking treatment.

### Knowledge and perception

The proportion of delay in seeking treatment was significantly high among participants who lacked information about TB. A person that is not educated about TB will not have information about prevention strategies, cure rate, transmission concerns and treatment options. As a consequence they might delay seeking diagnosis and treatment. This study is in line with a study conducted in North Wollo, Ethiopia which revealed that having good knowledge about TB is a prerequisite for early seeking of medical care [18]. This shows that concerned bodies should work in creating awareness about TB transmission, treatment and prevention.

Participants whose perceptions that TB cannot be cured were more likely to delay seeking treatment. Individuals who perceive their illness is not cured will become despondent and may not visit health facilities. In contrast to our study, a report from Uganda revealed that patients those who thought that TB could be cured were more likely to delay seeking treatment because their awareness makes them reluctant [34].

In our study, a significant number of TB patients delay seeking treatment in Adama, Ethiopia. This has several implications: because of the delay in seeking treatment the patient’s condition will worsen; the patient could disseminate the disease to family household members and to the community in general. According to our study, female sex, low income, lack of information on the disease and cure rate was factors that contributed to a delay in seeking treatment. Therefore, focusing on factors which prevent TB patients from seeking treatment will minimize the dissemination of the disease in the community.

### Limitations of the study

The data for this study was collected at health facilities when the patients presented with sign and symptoms. The patients may fail to remember the exact time. Therefore, it is prone to recall bias. Since convenience sampling technique was used, this study is prone to selection bias. As a result the finding of this study will not represent the target population. Since the study was cross sectional, it was difficult to establish temporal relationship between cause and effect.

## Conclusion

In this study we identified a substantial delay in TB treatment seeking among TB suspected patients. Nearly half of pulmonary tuberculosis suspected patients delayed seeking treatment at public health facilities. Factors such as: being female, low income, household size of five and above, lack of information about TB, and perception that TB cannot be cured were significantly associated with delay in seeking treatment. This study attempted to assess factors and behaviors that would delay seeking treatment from patients’ perspective only. We recommend further study to identify others factor that could contribute to delay in seeking TB treatment.

## Data Availability

All relevant data are available within the paper.

## Abbreviations

DOTS: Direct observed therapy
HIV: Human Immuno-deficiency Virus
TB: Tuberculosis
